# To be or not to be oxidized: A case study of olivine behavior in the fusion crust of ureilite A 09368 and H chondrites A 09004 and A 09502

**DOI:** 10.1111/maps.13284

**Published:** 2019-03-28

**Authors:** Lidia Pittarello, Akira Yamaguchi, Julia Roszjar, Vinciane Debaille, Christian Koeberl, Philippe Claeys

**Affiliations:** ^1^ Department of Lithospheric Research University of Vienna Althanstraße 14 A‐1090 Vienna Austria; ^2^ National Institute of Polar Research 10‐3 Midori‐cho Tachikawa Tokyo 190‐8518 Japan; ^3^ Department of Polar Science School of Multidisciplinary Science SOKENDAI (The Graduate University for Advanced Studies) Tokyo 190‐8518 Japan; ^4^ Natural History Museum Vienna Burgring 7 A‐1010 Vienna Austria; ^5^ Laboratoire G‐Time (Géochimie: Traçage isotopique, minéralogique et élémentaire) Université Libre de Bruxelles Av. F.D. Roosevelt 50 1050 Brussels Belgium; ^6^ Analytical, Environmental and Geo‐Chemistry (AMGC) Vrije Universiteit Brussel Pleinlaan 2 B‐1050 Brussels Belgium

## Abstract

Meteorite fusion crusts are quenched melt layers formed during meteoroid atmospheric entry, mostly preserved as coating on the meteorite surface. Antarctic ureilite Asuka (A) 09368 and H chondrites A 09004 and A 09502 exhibit well preserved thick fusion crusts, characterized by extensive olivine crystallization. As olivine is one of the major components of most meteorites and its petrologic behavior is well constrained, it can be roughly considered as representative for the bulk meteorite. Thus, in this work, the evolution of olivine in fusion crusts of the above‐listed selected samples is investigated. The different shape and chemistry of olivine crystallized in the fusion crust, both as overgrown rim on relic olivine clasts and as new crystals, suggest a general temperature and cooling rate gradient. The occurrence of reverse and oscillatory zoning in individual olivine grains within the fusion crust suggests complex redox reactions. Overall, the investigated fusion crusts exhibit a general oxidation of the relatively reduced initial material. However, evidence of local reduction is preserved. Reduction is likely triggered by the presence of carbon in the ureilite or by overheating during the atmospheric entry. Constraining these processes provides a potential analog for interpreting features observed in cosmic spherules and micrometeorites and for calibrating experiments and numerical models on the formation of fusion crusts.

## Introduction

Meteorite fusion crust is a blackish layer that generally encloses meteorites and that consists of quenched melt formed by heating due to friction with air molecules during the atmospheric entry of meteoroids, which occurs at high velocities (typically >10 km s^−1^; e.g., Love and Brownlee 1994). The melt produced at the surface of meteoroids is largely ablated, possibly as droplets forming spherules (meteorite ablation spherules), but commonly a thin layer is preserved, as one of the typical features of meteorites in the field (e.g., Genge and Grady 1999). The thickness of the fusion crust is controlled by the composition of the meteoroid (i.e., stony versus iron); by the spinning of the meteoroid during the atmospheric entry; and by other factors, such as the entry angle and velocity. The rotation of the meteoroid during the flight can cause local accumulation of melt on one side, in shadow with respect to the entry direction, and a comparatively thinner coat on the opposite side. Fusion crust forms on all kinds of meteorites, but generally it is thicker on iron meteorites than on stony chondrites, possibly due to the high heat conductivity of metal (El Goresy and Fechtig 1967). The resulting thickness can reach several mm. According to these authors, the formation of fusion crust in iron meteorites is accompanied by an apparent enrichment in Ni and Co in the quenched melt, likely due to oxidation of the original material. The readiness of Fe to be oxidized with respect to Ni and Co is well established (e.g., Rubin et al. 1988). Similar results, although complicated by the presence of sulfur, were described by Horstmann et al. (2013). In stony meteorites, even though fusion crust results from melting of the outer part of the meteoroid, the bulk composition of the melt slightly differs from that of the original material (Genge and Grady 1999 and references therein). This has been attributed to melt evolution (similar to magmatic fractional crystallization) or to influence of the local mineral composition (Thaisen and Taylor 2009). However, a “basaltic” melt composition has been obtained in experiments also from granitic rocks (Brandstätter et al. 2008), suggesting that the final composition is controlled by flash melting processes (i.e., individual phase melting point) rather than by the original bulk composition. The difference in bulk composition of the fusion crust with respect to the bulk meteorite mostly consists in enrichment of FeO, TiO_2_, Al_2_O_3_, Na_2_O, MnO, and K_2_O in the melt, with local variations reflecting the spatially diverse mineralogy of individual samples, as no real mixing occurred (Thaisen and Taylor 2009). The melt rim observed on the surface of some micrometeorites has been compared to meteorite fusion crust, as it exhibits depletion in highly to moderately volatile elements and relative enrichment in Fe, Ni, and Mn with respect to the unmelted cores (Genge 2006).

There is renewed interest in meteorite fusion crust because it could serve as a potential analog for chondrule formation, by comparison between meteorite ablation spherules, cosmic spherules, and chondrules, implying a similar melting and ablation process (Genge 2000), and due to similarities in Fe and O isotope composition between meteorite fusion crust and chondrules (Hezel et al. 2015). In addition, as already proposed by El Goresy and Fechtig (1967) and Genge and Grady (1999), the processes operating in the newly forming fusion crust and their relationship with the unaffected interior of the meteorite replicate those occurring in cosmic spherules during the atmospheric entry, and thus provide a key to understanding the formation of micrometeorites. However, due to the large size of meteoroids in comparison with micrometeorites, strong thermal gradients develop through the meteoroid itself during atmospheric entry (e.g., Genge 2006). This limits a direct comparison between melting effects in micrometeorites and in actual meteorites.

**Figure 1:**
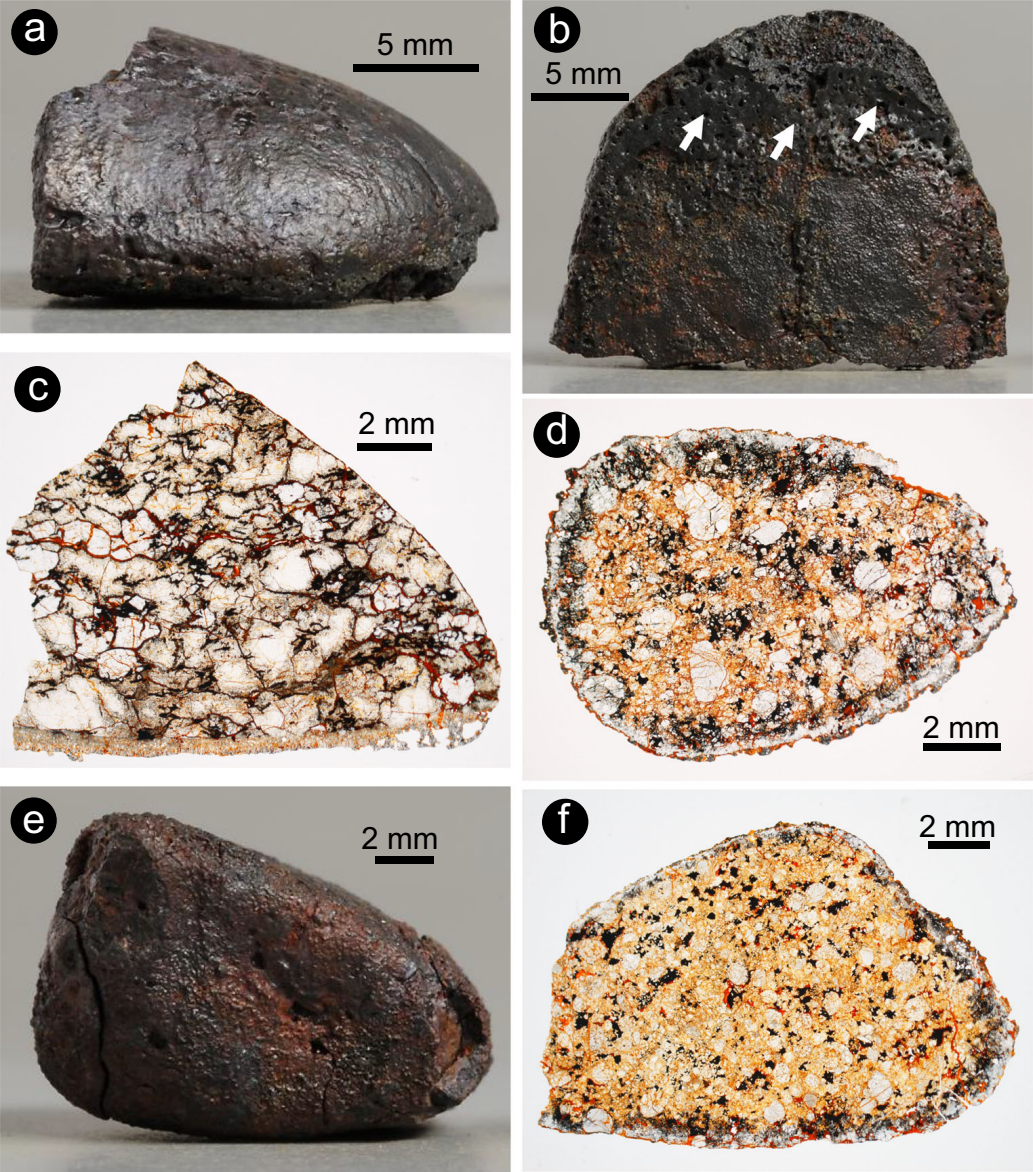
Meteorite samples and respective petrographic thin sections. a–c) Ureilite A 09368. a, b) Full sample photographs showing the flattened shape and the accumulation of melt on one side of the sample. c) Optical image of the thin section obtained from a chip of A 09368. The fusion crust is particularly thick (up to 1.5 mm) on the bottom side of the chip. d) Thin section scan of H5 chondrite A 09004. The fusion crust covers all the sides of the sample with rather constant thickness. e) Full sample photograph of H5 chondrite A 09502, showing a relatively thick fusion crust. f) Thin section photograph of a chip from (e), showing the relatively constant thickness of the fusion crust all around the sample. (Color figure can be viewed at wileyonlinelibrary.com.)

In this work, we focus on olivine as a representative meteoritic material, as olivine occurs not only as a common rock‐forming mineral in meteorites but also the major component in the fusion crusts of the selected samples. Ureilites are peculiar stony meteorites, mostly consisting of olivine and pyroxene, with a certain amount of carbon, whose origin is still debated (e.g., Mittlefehldt et al. 1998; Goodrich et al. 2014). Due to the presence of carbon (e.g., Hezel et al. 2008; Nagashima et al. 2012), ureilites represent relatively reduced planetary material. In the comprehensive work by Genge and Grady (1999), two ureilites were investigated (Novo‐Urei and Goalpara) and both exhibit a thin fusion crust, consisting entirely of quenched melt. On the other hand, the investigated sample A 09368, a ureilite collected during the 2009–2010 joint Japanese‐Belgian mission to Antarctica (Yamaguchi et al. 2014), locally exhibits a thick fusion crust, mostly consisting of coarse‐grained olivine crystals embedded in a glassy groundmass. Two equilibrated (petrologic type 5) H chondrites, A 09004 and A 09502, also from the shared Japanese‐Belgian Antarctic collection, show fusion crusts with very similar features to those in ureilites and were, therefore, selected for comparison (Fig. 1), although the original mineralogy is quite different between ureilites and chondrites (e.g., Hutchison 2004). Indeed, ureilites and H chondrites share a relatively reduced original chemical composition (e.g., Rubin et al. 1988; Jarosewich 1990), thus providing the best opportunity for investigating the possible effects of interaction with atmospheric oxygen. The evolution of olivine, from melting to growth and crystallization during quenching and its change in composition is then interpreted in the framework of fusion crust cooling rate and redox processes.

## Methods

Polished thin sections (30 μm thick) of the investigated samples, provided by the National Institute of Polar Research (NIPR), Tachikawa, Japan, have been studied with optical and electron microscopes at the NIPR. Microimaging was performed with a JEOL JSM‐7100F field‐emission scanning electron microscope (SEM), equipped with an energy dispersive detector (EDS), operated at 15 kV acceleration voltage, approximately 300 pA beam current, and 10 mm working distance.

Compositional data for the investigated mineral phases were collected with a JEOL JXA‐8200 electron microprobe (EMPA) at the NIPR, equipped with five wavelength‐dispersive spectrometers and one EDS. Operative conditions were 15 kV acceleration voltage, 12 nA beam current, and with a fully focused beam. For quantification, a ZAF correction was applied. Detection limits for major elements are 91 μg g^−1^ for Si, 137 μg g^−1^ for Ti, 44 μg g^−1^ for Al, 228 μg g^−1^ for Cr, 215 μg g^−1^ for Fe, 86 μg g^−1^ Mn, 52 μg g^−1^ for Mg, 80 μg g^−1^ for Ca, 40 μg g^−1^ for Na, and 165 μg g^−1^ for Ni. Natural and synthetic materials obtained from C.M. Taylor Company were used as mineral reference material. The composition of olivine ([Mg,Fe]_2_SiO_4_) is expressed as fayalite Fe_2_SiO_4_ (Fa) mole% (Mg/[Mg+Fe] × 100). The mineral abbreviations Ol for olivine and Fa for fayalite, according to Whitney and Evans (2010), are used throughout this manuscript.

The thin sections from the H chondrites were additionally analyzed at the Natural History Museum Vienna (Austria) for cathodoluminescence (CL) with a JEOL JSM 6610‐LV SEM equipped with a Gatan MonoCL (MonoCL4) system. The operating conditions for obtaining all SEM‐Mono CL images were 15 kV accelerating voltage and 1.2 nA beam current. CL images were recorded at a working distance of ~10 mm and at room temperature in the wavelength range of 200–800 nm (panchromatic).

## Results

### Petrographic Observations

The ureilite A 09368 (Fig. 1) has a broken, flattened hemispherical shape, and is almost completely coated by fresh fusion crust, which appears blackish on the hand sample. The more convex side of the flattened hemisphere exhibits a thin fusion crust, with surface features resembling regmaglypts (Fig. 1a). The more flattened side exhibits a thicker fusion crust (Fig. 1b). The polished thin section was cut from this side, where the fusion crust has an average thickness of 1 mm. In thin section, the fusion crust appears not to consist of solely quenched melt, but rather to contain large crystals of olivine that have crystallized from the melt (Fig. 1c). The inner part of the meteorite, unaffected by melting, consists of a coarse‐grained, clast‐supported ureilite olivine breccia comprising mainly olivine grains with angular shape and average mm grain size and local rare occurrence of pyroxene (tens of μm).

Sample A 09004 has a roughly square shape and seems to be homogeneously coated by a thin layer of fusion crust (Fig. 1d). Sample A 09502 has a more rounded shape and seems to have a thicker fusion crust on one side (Fig. 1e). In thin section, this observation is confirmed, with a maximum thickness of the fusion crust of approximately 1 mm (Fig. 1f). The interiors of both chondrite samples, not affected by melting, are consistent with the common characteristics for petrologic type 5 H chondrites (e.g., Weisberg et al. 2006). Chondrite A 09004 contains rare chondrules and individual large olivine and pyroxene crystals (up to 2 mm) in a coarse‐grained crystalline matrix. Chondrite A 09502 exhibits more abundant small (about 1 mm) relic chondrules in a coarse‐grained crystalline matrix. The metal content is high in both samples, consistent with the classification as H chondrites, but metal is relatively oxidized due to terrestrial weathering, despite the fact that these meteorites were collected in Antarctica, where oxidation is relatively limited due to the low temperature in comparison with hot desert environments. Compared to the ureilite, chondrites have a heterogeneous mineral content in direct contact with the fusion crust. In the chondrites, olivine crystals are equilibrated (zoning is absent) in the interior of the meteorite, as expected in type 5 ordinary chondrites, which were exposed to temperatures as high as 600–900 °C (Huss et al. 2006). Nevertheless, the features observed in the fusion crust of these two chondrites are very similar to those present in the fusion crust of the investigated ureilite.

**Figure 2:**
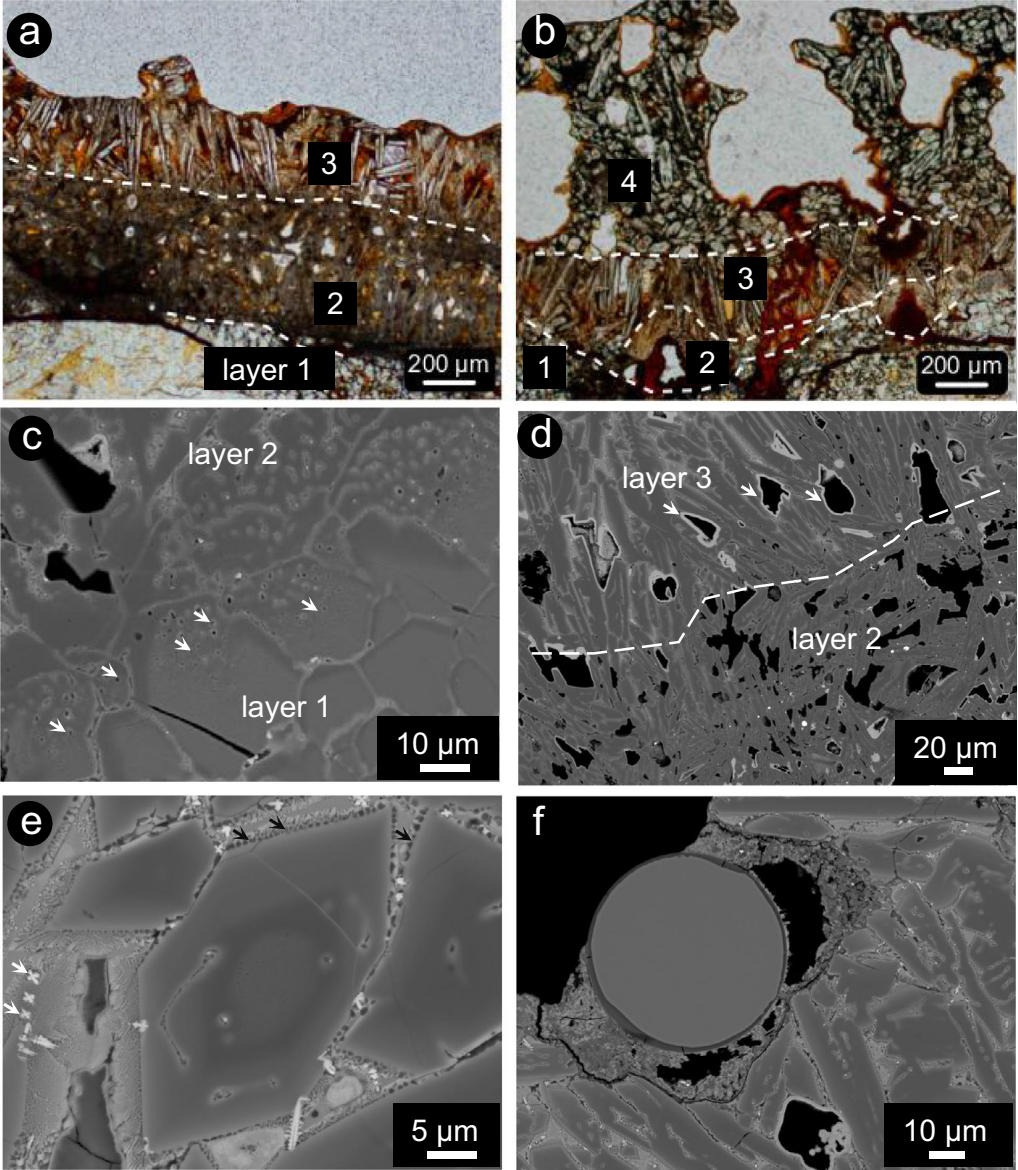
Fusion crust layers in ureilite A 09368. a) The three layers as described in the text. Plane‐polarized light. b) Area of the fusion crust, where a fourth layer is visible, consisting of coarse‐grained olivine. Plane‐polarized light. c) The transition between layer 1 and 2, marked by a darker color in BSE‐SEM images and the appearance of vesicles in olivine (white arrows). BSE‐SEM image. d) Transition between layer 2 and layer 3. Note the abundant vesiculation in layer 2 and the bright rim coating vesicles (white arrows) in layer 3. BSE‐SEM image. e) Detail in layer 2, showing evidence of liquid immiscibility in the interspace between olivine crystals (black arrows), chemical zoning in olivine, and the presence of skeletal magnetite (white arrows). BSE‐SEM image. f) “Spherule” trapped in the outer part of the fusion crust. BSE‐SEM image. (Color figure can be viewed at wileyonlinelibrary.com.)

Within the fusion crust of the ureilite, three domains can be recognized based on the morphology of olivine. These layers are numbered from the interior of the meteorite to the outer part of the fusion crust from layer 1 to layer 3, plus a fourth layer that occurs only locally (Figs. 2a and 2b). Layer 1 is characterized by angular olivine, with finer grain size than in the interior of the meteorite (10–20 μm average size with respect to mm; Fig. 2c). Olivine grains present a rim with darker color in backscattered electron (BSE) images than the host crystal and contain tiny (<1 μm in diameter), round vesicles. Between olivine grains, interstitial melt, with bright color in BSE‐SEM images, occurs (Fig. 2c). At the transition to the layer 2, chemical zoning is sharp, within individual olivine grains, forming a rim that has darker gray color in BSE‐SEM images than the core, suggesting lower Fa content than in the olivine in the interior of the meteorite.

Layer 2 contains fragments of relic olivine with extensive overgrowth, suspended in a partially crystallized melt, with abundant vesiculation (Fig. 2d). Relic olivine from the interior of the meteorite is preserved at the core of olivine grains. These cores exhibit overgrown rims with subhedral shape (Fig. 2e). The core of some olivine grains appears rounded and shows similar vesiculation as in the fractured olivine in layer 1. The overgrowth is characterized by at least two different BSE gray tones. The first rim has a darker color (darker in BSE‐SEM images) than the core, as observed in layer 1, whereas the outer rim contains vesicles and has a brighter color in BSE‐SEM images, suggesting a higher Fa content than in the first rim. New subhedral to euhedral olivine crystals and overgrown rims are suspended in a glassy groundmass, where small (μm‐sized) skeletal magnetite occurs (Fig. 2e). In the groundmass, silica‐rich droplets surrounding olivine in a silica‐poor quenched melt suggest liquid immiscibility (Fig. 2e).

Layer 3 consists of olivine microlites, acicular crystals of secondary olivine, showing hopper and chain morphology, up to 100 μm in size and embedded in a glassy groundmass (Figs. 2d–f). These large olivine crystals exhibit zoning toward the rim. Skeletal magnetite also occurs and the vesicles, whose shape is determined by that of olivine crystals, are rimmed by phases that possess bright whitish color in BSE‐SEM images, likely Fe‐oxides (Fig. 2d). In the outer part of this layer, a melt spherule with a diameter of about 60 μm occurs (Fig. 2f), with a homogeneous composition that is very similar to that of the average bulk composition of ureilites (after Jarosewich 1990), likely representing a meteorite ablation spherule, quenched before being detached (e.g., Genge and Grady 1999).

Layer 4 contains subhedral olivine crystal aggregates with average grain size of ~100 μm (Fig. 2b) and abundant round vesicles. Layer 4 is only observed at the margin of the whole sample in the investigated thin section, where the preserved fusion crust reaches its maximum thickness. The thickness of the layers varies considerably within the sample. The boundary between the first and the second layer is quite sharp (Fig. 2c), whereas the transition between the second and the third layer is more gradational (Fig. 2d). The abundance and the size of vesicles increase toward the outer part of the fusion crust.

**Figure 3:**
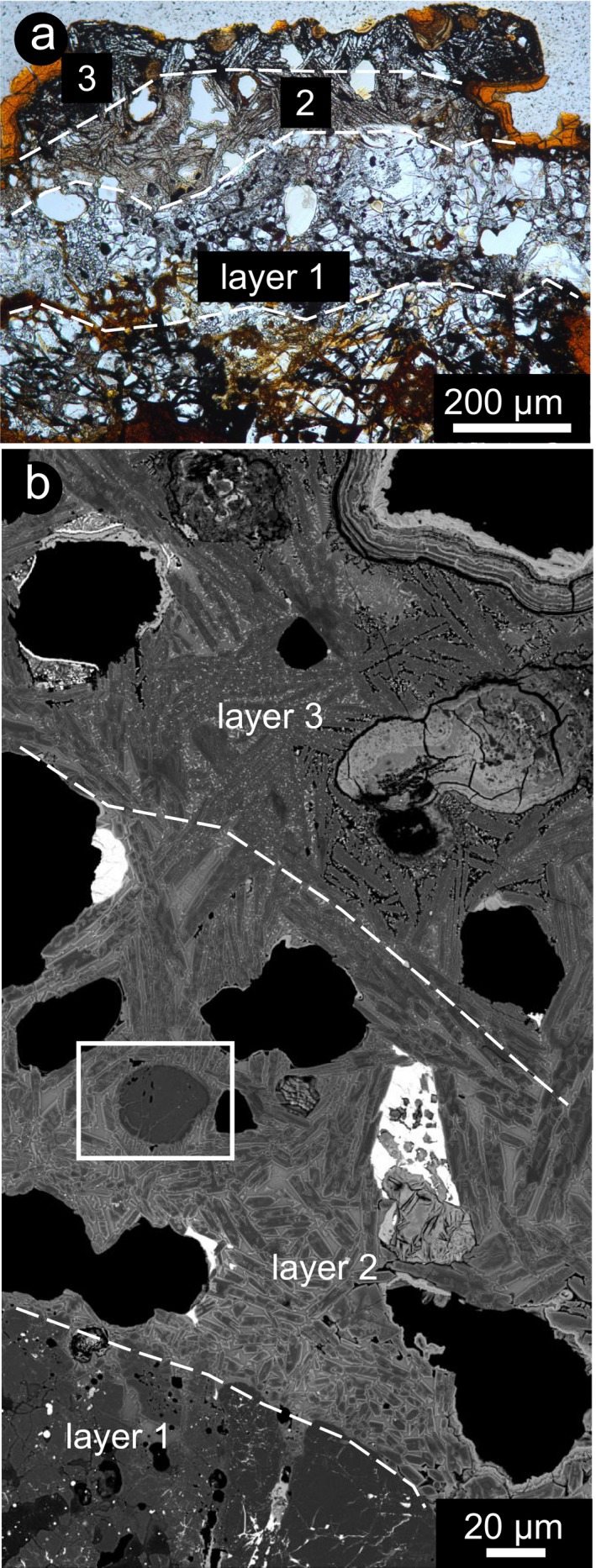
Fusion crust layers in H5 chondrites A 09004 and A 09502. a) Thick portion of fusion crust with the three layers described in the text. Sample A 09004. Plane‐polarized light. b) Composite image through the fusion crust of sample A 09502, showing the three layers. Note the large vesicles in layer 2 and the presence of skeletal magnetite (bright spots) in layer 3. BSE‐SEM image. (Color figure can be viewed at wileyonlinelibrary.com.)

**Figure 4:**
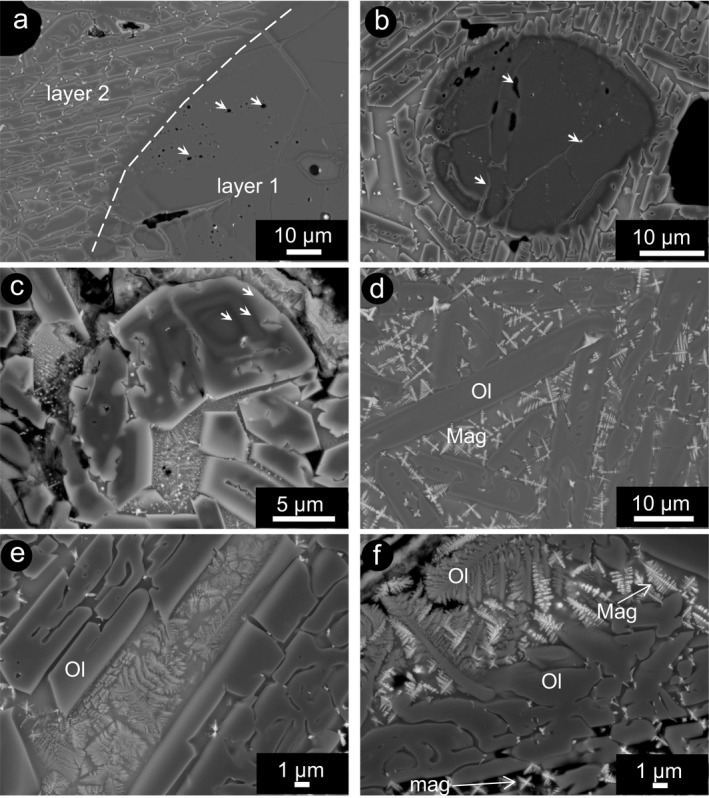
BSE‐SEM images of olivine and other phases in the fusion crust of H5 chondrites A 09004 and A 09502. a) Transition from layer 1 to layer 2. Note the appearance of fine‐grained round vesiculation within the olivine. Sample A 09004. b) Sintering of relic fragments of olivine, with interstitial melt, vesicles and bright inclusions marked with arrows. Sample A 09502. c) Oscillatory zoning in olivine growth in the melt and hopper olivine. Sample A 09502. d) Olivine microlites, locally showing hopper and chain shapes (Donaldson 1976), with bright margins and skeletal magnetite in the groundmass. Sample A 09004. e) Olivine hopper microlites and incipient dendritic crystallization of second generation phases in the melt. Sample A 09502. f) Interstitial melt between complex shaped olivine showing swallow‐tail olivine and skeletal magnetite, in the outer layer of the fusion crust. Sample A 09502. The mineral abbreviations Ol for olivine and Mag for magnetite, according to Whitney and Evans (2010) are used.

Also in case of the H chondrites, the fusion crust exhibits a well‐defined layering, even though simpler with respect to that in the fusion crust of the ureilite. Layer 1 consists of angular olivine and pyroxene, layer 2 consists of olivine acicular crystals embedded in quenched melt, relic olivine with secondary olivine overgrowth and locally skeletal magnetite, and layer 3 is different from layer 2 because of the progressive coarser grain size of the olivine crystals and increasing crystallization of the melt (Figs. 3a and 3b). Locally, a melt pocket is present between layers 1 and 2. In detail, layer 2 shows progressively increasing grain sizes of secondary olivine toward the outer part of the fusion crust (from about 10 to about 100 μm), from melt/matrix‐supported to grain‐supported fabric, with decreasing vesicle abundance (Fig. 3b). At the transition between layer 1 and 2 and in olivine relics within layer 2, olivine exhibits vesiculation and a rim with relatively dark gray color in BSE‐SEM images (Fig. 4a), similar to what is observed in the ureilite. In the inner part of layer 2, relic olivine fragments occur, locally characterized by overgrowth, coalescence between grains and sintering (Fig. 4b). In the case of sintering, original grain boundaries are recognizable by the presence of a film of melt and trails of inclusions (Fig. 4b). Locally, relic olivine has a rough round shape at the core, with oscillatory chemical zoning (Fig. 4c). Alternating gray intensities in BSE‐SEM images within olivine indicates cyclical changes in the Fe/Mg ratio in the newly formed olivine. The outermost part of the growth rim is always extremely bright in BSE‐SEM images, indicating higher Fe/Mg ratio than in the inner part, corresponding to what is defined as normal zoning in magmatic processes (Figs. 4c–f). The groundmass progressively changes from melt‐supported (mostly homogenous quenched melt; Fig. 4b), to melt with skeletal magnetite (Fig. 4d), and to completely microcrystalline groundmass, with dendritic crystals of likely Fe‐rich olivine or pyroxene and skeletal magnetite (Figs. 4e and 4f), toward the outer margin of the fusion crust.

**Figure 5:**
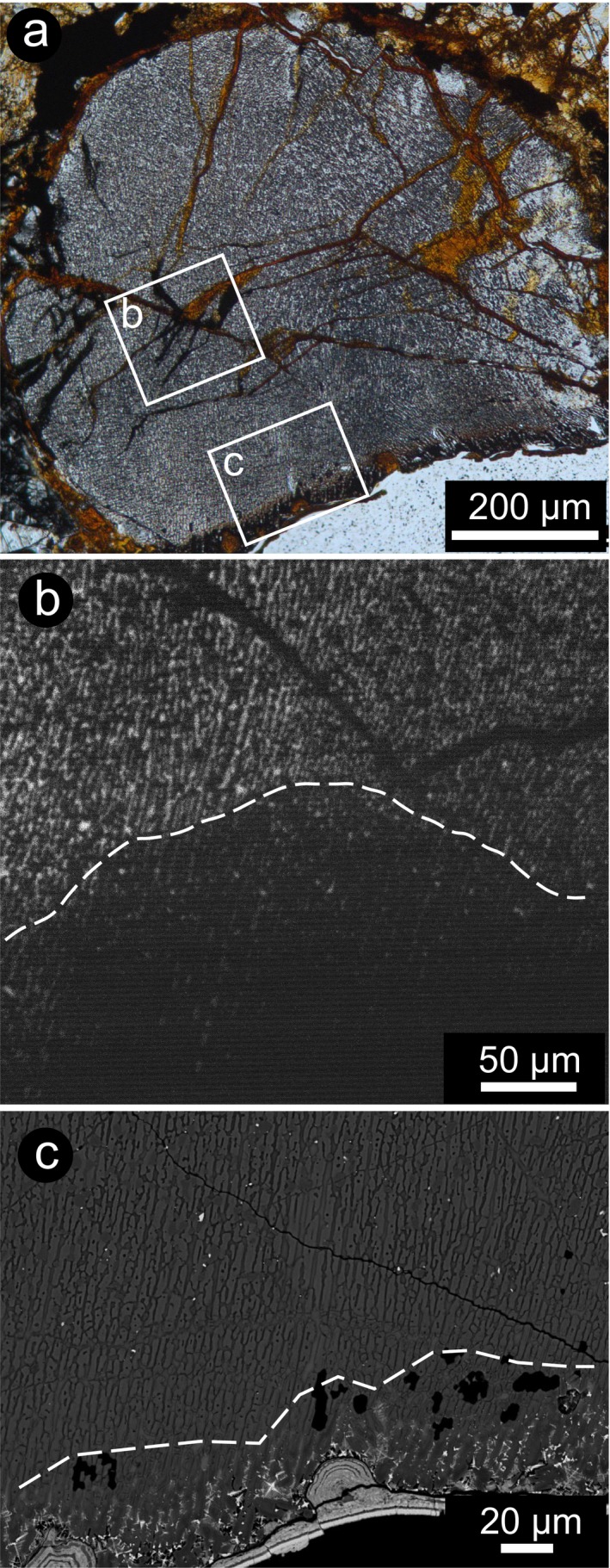
Depth of the thermal effect on a relic chondrule next to the meteorite margin. Sample A 09502. a) Microphotograph of the chondrule. b) SEM‐CL monochromatic image of a detail in the chondrule. The transition from luminescent feldspathic mesostasis and quenched area is marked with a dashed line. c) Incipient melting at the outer margin of the chondrule, crystallization of skeletal magnetite, and appearance of amoeboid vesiculation. The apparent transition into the thermally affected portion is marked with a dashed line. BSE‐SEM image. (Color figure can be viewed at wileyonlinelibrary.com.)

Even though the overgrowth structures of olivine could not be investigated by CL as the high‐Fe content resulted in a quenched signal, this analytical technique applied to feldspathic mesostasis provides an unexpected evaluation of the extent (depth) of the thermal effect, far beyond optical resolution (Fig. 5) and mineralogical evidence (e.g., back‐transformation of high‐pressure phases into the low‐pressure polymorphs due to high temperature; Kimura et al. 2003). With the optical microscope, the fabric of a relic fine‐grained barred olivine chondrule in A 09502 seems altered only in the last 50 μm toward the margin (Fig. 5a). However, a pronounced decrease in luminescence of the crystallized feldspathic mesostasis inside the chondrule highlights the depths of such a thermal effect, which reaches >150 μm (Fig. 5b). This effect was not visible even in BSE‐SEM images, which show that the occurrence of vesicles, remelting, and fast crystallization of the rim, with the formation of skeletal magnetite, affect only the very outer part of the chondrule (40–50 μm).

### Olivine Chemistry in the Fusion Crust

**Figure 6:**
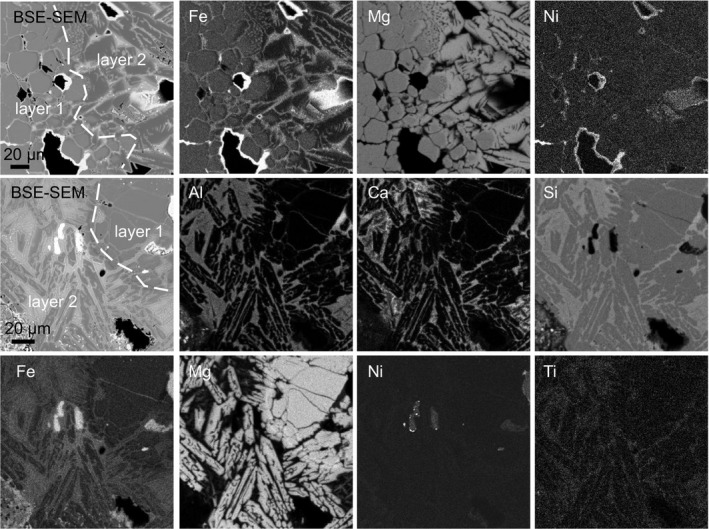
X‐ray element maps of the transition zone between layer 1 and 2 in the ureilite A 09368 (top row) and in the H chondrite A 09004 (bottom two rows). The original BSE‐SEM image is compared with individual element maps for selected elements. See text for explanation.

Olivine in the fusion crust of both A 09368 ureilite and H chondrites A 09004 and A 09502 occurs either with slightly lower or with higher Fa content than olivine in the interior of the meteorite (Fig. 6; Tables 1 and 2). These two cases are represented in both newly formed dendritic olivine and overgrown olivine on relic clasts. The general trend observed for the content of some minor elements is similar for both occurrences with respect to that for olivine from the interior of the meteorite. The transition from layer 1 to layer 2 is indeed marked by enrichment in MgO and apparent depletion in FeO (Fig. 6). The occurrence of Mg‐rich olivine is limited to thin growth rims in the fusion crust of H chondrites, and therefore this population is less represented than in the fusion crust of the investigated ureilite. The detailed behavior of minor elements cannot be appreciated in X‐ray element maps, but is obvious comparing spot analyses (Tables 1 and 2). On the other hand, X‐ray element maps show that Al, Ca, and Ti are preferentially contained in the melt surrounding olivine and that Ni and Fe are particularly concentrated in the rim of the vesicles.

**Table 1:** Olivine compositions in ureilite A 09368, from the interior of the meteorite to the outer part of the fusion crust. Sample A 09368. Electron microprobe data (wt%)

	Host meteorite Ol (avg #5)	Fusion crust Ol layer 1	Ol rim layer 1	Relic Ol core (avg #4)	Ol dark rim (avg #5)	Ol bright rim	Spherule (avg #2)	Avg ureilite*
SiO_2_	39.32 (16)	39.61	41.10	40.77 (28)	41.47 (30)	40.06	47.12 (12)	40.35
TiO_2_	bdl	bdl	bdl	bdl	bdl	0.03	0.17 (1)	0.07
Al_2_O_3_	bdl	bdl	bdl	bdl	bdl	bdl	4.13 (2)	0.20
Cr_2_O_3_	0.61 (5)	0.41	0.33	0.51 (10)	0.44 (13)	0.37	0.06 (5)	0.69
MgO	41.94 (15)	44.01	51.04	48.53 (53)	51.28 (96)	45.98	29.44 (6)	35.92
CaO	0.29 (3)	0.18	0.07	0.15 (9)	0.09 (7)	0.08	3.52 (1)	1.43
MnO	0.41 (1)	0.39	0.20	0.38 (16)	0.26 (13)	0.29	0.57 (1)	0.39
FeO	18.39 (55)	15.22	6.87	10.16 (62)	6.62 (99)	13.65	14.09 (5)	14.36
NiO	bdl	bdl	0.05	bdl	0.05 (7)	0.09	bdl	0.16
Na_2_O	bdl	bdl	bdl	bdl	bdl	bdl	bdl	0.05
Total	100.97	99.82	99.66	100.50	100.23	100.55	99.13	
Fa	19.7 (5)	16	7	10.5 (7)	6.7 (9)	14	‐	

avg = average of the indicated number (#) of spot analyses, with standard deviation; Avg ureilite* = average ureilite composition after Jarosewich (1990), only absolute content of selected elements (tot < 100); bdl = below detection limit.

**Table 2:** Olivine composition of H chondrites A 09502 and A 09004, from the interior of the meteorite to the outer part of the fusion crust. Electron microprobe data (wt%)

	Host meteorite Ol in A 09502 (avg #3)	Fusion crust Ol 1 (avg #2)	Fusion crust Ol 2 (avg #2)	Host meteorite Ol in A 09004 (avg #4)	Fusion crust ol
SiO_2_	39.24 (47)	40.10 (36)	38.42 (91)	39.71 (38)	40.65
TiO_2_	0.07 (5)	bdl	0.05	bdl	bdl
Al_2_O_3_	0.07 (10)	0.10 (6)	0.63 (31)	bdl	0.09
Cr_2_O_3_	bdl	0.21 (12)	0.28 (6)	bdl	0.34
MgO	42.65 (66)	45.11 (99)	32.56 (38)	43.73 (33)	47.55
CaO	0.05 (4)	0.10 (5)	0.50 (29)	0.06 (9)	0.09
MnO	0.44 (2)	0.28 (9)	0.31 (2)	0.45 (1)	0.20
FeO	17.60 (57)	14.05 (136)	26.05 (56)	17.23 (21)	11.86
NiO	0.05 (5)	0.28 (23)	0.47 (4)	bdl	0.66
Na_2_O	bdl	bdl	0.12 (2)	bdl	bdl
Total	100.16	100.22	99.36	101.18	101.43
Fa	18.7 (7)	14.8 (15)	30.9 (7)	18.0 (3)	12

avg = average of the indicated number (#) of spot analyses, with standard deviation; fusion crust Ol 1 = first dark layer of olivine overgrowth; fusion crust Ol 2 = outer overgrowth rim; bdl = below detection limit.

**Figure 7:**
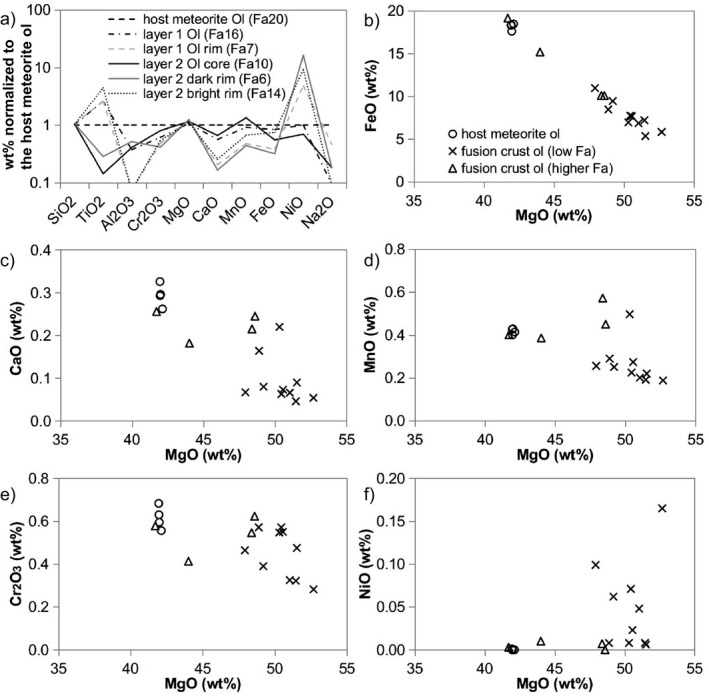
Olivine chemical composition in the fusion crust of ureilite A 09368. In the spider diagram (a), the major element composition of fusion crust olivine is normalized to the composition of the olivine in the interior of the meteorite (host meteorite Ol with Fa_20_). The olivine contents of individual important elements (as oxides) are plotted versus MgO (b–f). Circles represent olivine in the interior of the meteorite (host meteorite ol) and the population olivine in the fusion crust is divided by the Fa content.

**Figure 8:**
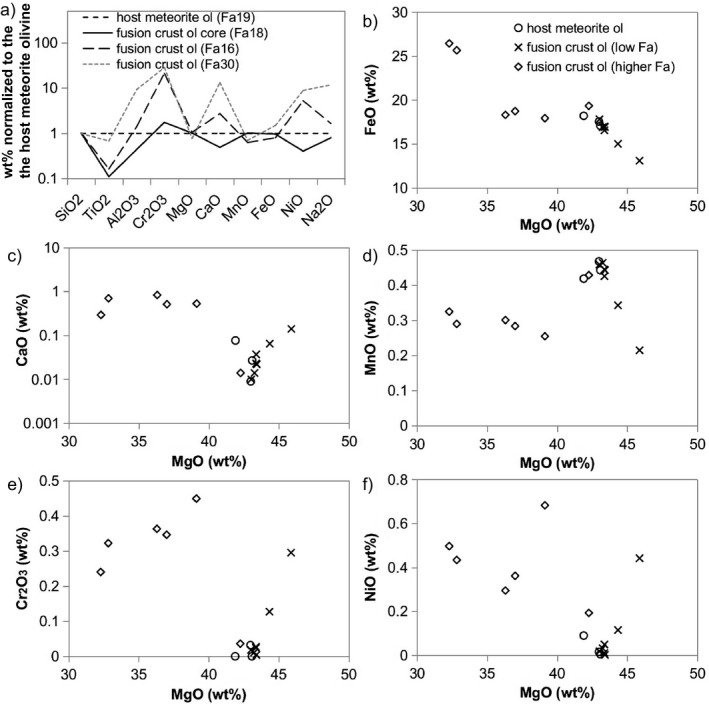
Olivine chemical composition in the fusion crust of ordinary chondrite H5. Microprobe analysis from sample A 09502. As in Fig. 7, the major element composition of fusion crust olivine normalized to the composition of the olivine in the interior of the meteorite (host meteorite Ol with Fa19) is shown in the spider diagram (a). The olivine contents of individual important elements (as oxides) plotted versus MgO are shown in diagrams (b–f).

Specifically, newly crystallized olivine grains in the ureilite fusion crust seem enriched in Ni and depleted in Ca and Cr, in comparison with the olivine in the interior of the meteorite (Fig. 7). In contrast, newly crystallized olivine in the fusion crust of H chondrites seems to be enriched in Al, Cr, Ca, and Ni, but depleted in Mn and Ti (Fig. 8). The core of relic olivine in layer 2, as well as the fractured olivine crystals in layer 1, characterized by the occurrence of diffuse vesiculation, show small but detectable changes in chemical composition with respect to the olivine in the interior of the meteorite (Table 2). The relic olivine seems to be depleted in Ti, Al, Ca, and Ni, and only slightly enriched in Cr. However, Fe and Mg contents are consistent with those in the olivine in the interior of the meteorite (Figs. 7 and 8; Tables 1 and 2).

In case of zoning in the overgrown olivine in layer 2, the Mg‐enriched rim directly in contact with the relic core is even more enriched in MgO than the core itself in comparison with the olivine in the interior of the meteorite, whereas the bright rim exhibits significant enrichment in FeO and NiO (Tables 1 and 2). Generally, the trend consists of a progressive enrichment in MgO and NiO in the firstly formed olivine, compensated by a relative depletion in FeO, CaO, and MnO. For further olivine crystallization, in the H chondrites, new olivine is progressively enriched in Fe and NiO, other than in CaO and Cr_2_O_3_ with respect to the olivine in the interior of the meteorite (Figs. 7 and 8). The extreme enrichment in CaO in the Fe‐rich olivine in the fusion crust of H chondrites (Fig. 8) might be apparent, due to analytical contamination from the neighbor melt, considering the limited thickness of the Fe‐rich olivine rim and the interaction volume of the electron microprobe beam. In the ureilite fusion crust, only the very outer rim of new olivine has higher FeO content than the olivine in the interior of the meteorite. However, in many cases, this rim is thinner than the spatial resolution of the EMPA and, thus, the resulting measurements are all contaminated by the groundmass. Considering the thickness of the described features, further analyses by laser ablation inductively coupled plasma–mass spectrometry and secondary ion mass spectroscopy (SIMS), which allow accurate detection of minor and trace elements, are not suitable, as the general spatial resolution of these techniques is higher than 25 μm (unless NanoSIMS is used). Local variations in the secondary olivine would, therefore, not be detectable.

## Discussion

### Cooling Rate(s) in the Fusion Crust

Numerical modeling of the cooling of meteorite fusion crust includes too many unknown variables and, therefore, has never been successfully performed. Nevertheless, information is provided by the occurrence of large vesicles, suggesting degassing (e.g., fusion crust of a carbonaceous chondrite [Genge and Grady 1999], of lunar meteorites [e.g., Joy et al. 2006], and in cosmic spherules [Genge 2017]). According to numerical modeling for cosmic spherules, for low peak temperature, vesicle migration within the melt is mostly controlled by the melt viscosity, whereas at high peak temperature (superheating, like those experienced in the fusion crust), the migration and coalescence of vesicles is controlled by the dynamic processes during transit through the Earth's atmosphere (Genge 2017). This implies that due to fast cooling, the vesicles in the fusion crust are quenched and stop migrating relatively soon after formation. This is confirmed by this study, where most of the vesicles appear to be trapped within layer 2 (Figs. 2a and 3).

In the investigated samples, layer 1 in the fusion crust exhibits intense fracturing. This is likely a response to high temperature, namely thermal fracturing, which is due to different thermal expansion coefficients between mineral phases characterized by different chemical compositions or even crystallographic orientations (see for example Osako et al. 2004 about the thermal diffusivity in olivine estimated along its three main directions).

The Mg‐Fe partitioning between olivine and melt is commonly used as geothermometer (e.g., Falloon et al. 2007) and generally the Mg content of olivine is correlated with the crystallization temperature. However, in the case of meteorite fusion crust, equilibrium between olivine and melt is never reached. This suggests that the Mg/Fe ratio in the secondary olivine in the fusion crust may reflect the very local conditions at a precise instant. In addition, the composition of the melt for thermometric calculations could not be determined due to incipient crystallization of phases other than olivine (e.g., Figs. 4e and 4f). There are also technical limitations in determining the composition of the thin rim in olivine microlites, in direct contact with the melt, as this rim is as thick as the interaction volume of the microprobe, increasing the risk of contamination from tiny inclusions and from the melt itself.

The shape and the grain size of the newly crystallized olivine can, however, provide a rough estimate of the crystallization temperature and the cooling rate in the fusion crust. A complete description of the different crystallization morphologies in olivine is presented by Donaldson (1976) and in the experimental work by Faure et al. (2003). All observed olivine morphologies in the investigated fusion crusts are consistent with a rapid cooling rate, likely between 1600 and 2000 °C h^−1^ (Faure et al. 2007). However, the actual rate cannot be determined from the morphology alone, because different shapes coexist in the same layer. The undercooling degree was likely different between layers in the fusion crust, considering the crystallization of secondary olivine (this does not apply to overgrowth). It seems that undercooling was lower in the inner part of the fusion crust than in the outer part, where also dendritic olivine occurs. This implies that olivine morphology is controlled by the undercooling degree and the availability of crystallization nuclei in the melt. The latter might be controlled in the frequency and distribution by the pre‐existing texture of the meteoroid. The whole crystallization occurred within minutes, as a longer crystallization time would have produced tabular crystals (Ni et al. 2014). The outer part of the fusion crust contains elongated olivine crystals that appear similar to barred olivine chondrules. Barred olivine forms from undercooling after complete melting at a temperature range of 1500–1700 °C and at a cooling rate between 500 and 2300 °C h ^−1^, depending on the relative composition of the melt and of the newly formed olivine (e.g., Lofgren and Lanier 1990). On the other hand, oxygen fugacity does not seem to affect olivine crystallization morphology (Donaldson 1976; Hammer 2006), and therefore, can be ignored in this evaluation.

The thickness and the composition of the overgrown olivine may also constrain the local cooling rate. Even though based on melt inclusions in olivine, the work by Newcombe et al. (2014) provides some insights into the dependence of growth distance of olivine with time. According to the model proposed by these authors, 1 μm growth requires only 150 s. However, the correlation between rim thickness and growth rate is not linear; with an assumed cooling rate of 2000 °C h^−1^ and in the temperature interval 1200–1000 °C, olivine growth is very rapid up to 400 μm, then it slows down almost to a stop. Obviously, olivine is supposed to crystallize during both cooling and re‐equilibration with the melt, which is also rapidly quenched in meteorite fusion crust. The assumed cooling rate is consistent with that determined by the shape of new olivine according to the work by Faure et al. (2003).

### Olivine Crystallization

Even though H chondrites consist of a variety of mineral phases, the local mineralogical composition in direct contact with the fusion crust seems not to affect the occurrence of olivine in the melt, in contrast with that described by Thaisen and Taylor (2009). This implies that crystallization of olivine originated from a relatively homogeneous melt, despite the short duration of the process. However, the observed homogenization might be due to melt accumulation or mixing phenomena during meteorites spinning.

In H chondrites, the main silicate components are olivine, pyroxene, and plagioclase. However, in the fusion crust, the only silicate to crystallize in the melt is olivine. This implies that olivine crystallization should have been determined by another process than just cooling of a mafic silicate melt. Jurewicz et al. (1991) and Longhi (1999) tried to explain the formation of differentiated basaltic meteorites, such as eucrites and angrites, from a general basaltic melt by redox reactions. Their results support the hypothesis that, upon melting and by reaction of metallic Fe with atmospheric oxygen, the melt is rapidly saturated in Fa‐rich olivine (with respect to the olivine not affected by high temperature modification) and in magnetite‐type spinel. This process leads to an undersaturation in pyroxene and feldspar. Thus, during cooling, olivine and magnetite crystallization occurs before that of pyroxene and plagioclase, regardless of the absolute melt temperature. Progressive oxidation of the metal contained in the meteorite is clearly linked also to the resulting enrichment in Fe and Ni in the new olivine in the fusion crust and to the precipitation of Fe‐ and Ni‐oxides coating the rim of vesicles (e.g., Fig. 6).

### Olivine Zoning in the Fusion Crust

In the investigated ureilite and H chondrites, fusion crusts exhibit similar microstructures, but slightly different chemical compositions of the new olivine. In the ureilite, olivine crystallized in the fusion crust is more magnesian (Fa_7_) than olivine in the interior of the meteorite (Fa_20_) and only the outer rim developed normal zoning, following fractional crystallization evolution, with a more fayalitic (Fa_14_) composition. In the H chondrites, new olivine in the fusion crust is generally more fayalitic (up to Fa_31_) and only locally a thin film (<1 μm) more forsteritic (Fa_15_) than the olivine in the interior of the meteorite (Fa_19_) occurs. However, a major difference between the two studied meteorite groups is that H chondrites contain a relatively high amount of metal (about 25 wt%) and ureilites may contain variable amounts of metal (from none, as in the case of A 09368, to chondritic value), but a relatively large amount of carbon (up to 4 wt%; Jarosewich 1990).

**Figure 9:**
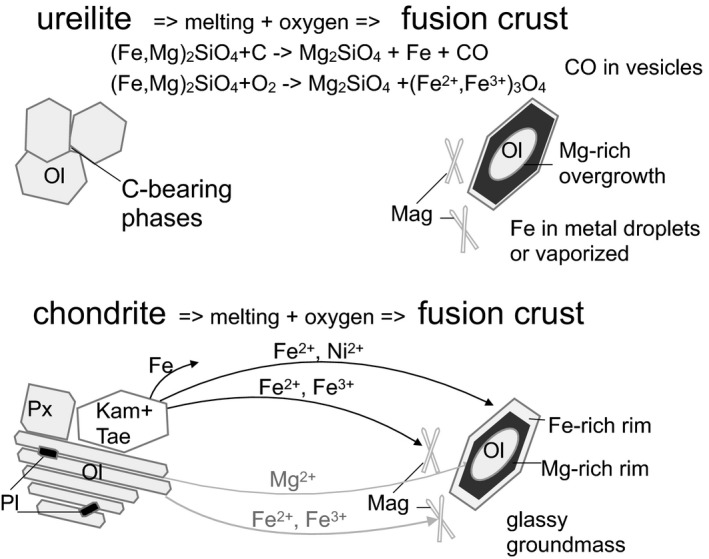
Cartoon representing the possible processes occurring in the fusion crust of the ureilite A 09368 and of the H chondrites A 09004 and A 09502, generating the reverse and oscillatory zoning in the olivine overgrowth.

Assuming that oxidation is responsible for the crystallization of olivine and magnetite, the occurrence of olivine rims in the fusion crust with higher Mg/Fe ratio than in the olivine in the interior of the meteorite requires another process to be explained. The composition of the secondary olivine might be also controlled by the oxygen fugacity (*f*O_2_) in the melt, as constrained in melting experiments for ordinary chondrite (Usui et al. 2015). However, effects induced by variation of *f*O_2_ are relatively negligible for the Fe^3+^/Fe^2+^ ratio in olivine (e.g., Putirka 2016). Other than a simple temperature effect, the occurrence of more magnesian olivine might, therefore, be related to (1) oxidation of Fe^2+^ into Fe^3+^, leading to crystallization of magnetite and of Mg‐rich olivine, and (2) initial reduction due to the presence of carbon in the host meteorite, forming metal droplets trapped in the newly crystallizing low‐Fa olivine. As stated above, carbon is present in negligible amounts in H chondrite, but is relatively enriched in ureilite. This would be consistent with the higher amount of low‐Fa content in the new olivine in the fusion crust of the ureilite with respect to H chondrite. Pyrolysis of carbon, producing reducing conditions, has been already considered to affect the fusion crust of carbonaceous chondrites (Genge and Grady 1999) and likely in carbonaceous micrometeorites (Genge 2006). Reduction caused by the presence of carbon has probably occurred in the early stage of melting, due to the increase of temperature, and was likely followed by oxidation of metal, when all the available carbon was degassed as CO or CO_2_. After the transformation of significant Fe into Fe^2+^, which might have been accelerated by the removal of Fe by vaporization, further oxidation into Fe^3+^ occurred. For comparison, oxidation processes in olivine due to atmospheric interaction in impact melts have been modeled by Sheffer and Melosh (2005). During decompression and cooling, the probability of bonding with oxygen of the vaporized material changes with temperature, following a complex pattern. As this probability is higher in the vapor than in the liquid, removal of vapor induces chemical reduction in the liquid. This process explains the formation of iron metal droplets in micrometeorites, without the need of a reduction agent. As a consequence, progressive ablation of the vapor phase might have caused temporary reduction in the melt and, therefore, crystallization of forsteritic olivine. A cyclical repetition of these processes (oxidation of metal, vaporization of Fe, and pyrolysis of carbon) due to progressive melting could explain the oscillatory zoning of overgrown olivine in the fusion crust. These processes are summarized in Fig. 9.

Other than vaporization of highly volatile elements, which are generally present in small amounts in olivine, an alternative explanation of the observed vesiculation in olivine at the transition between layer 1 and 2 and in the core of relic clasts in layer 2 could be related to a shock pressure, which could be reached during atmospheric entry. Porosity and melting in olivine are observed for shock pressure as high as 80 GPa in experiments and also attributed to high *f*O_2_ at pressure exceeding 55 GPa, inducing oxidation of Fe^2+^ to Fe^3+^ (Bauer 1979). Even though these dated experiments are not fully representative of shock behavior of olivine, the observation of oxidation processes is quite interesting and confirms that olivine with lower Fe/Mg ratio can also be obtained by extreme oxidation, rather than by reduction.

### Is the Fusion Crust Representative for Cosmic Spherules?

Focusing only on olivine, the microstructures observed in cosmic spherules (Koeberl and Hagen 1989; Taylor and Brownlee 1991) are very similar to those presented here, recalling the occurrence of so‐called porphyritic olivine (PO), relic grain‐bearing, and amoeboid olivine aggregate, according to the description in Yada et al. (2005). The most common feature in the above‐mentioned types of spherules is the preservation of a forsteritic core in olivine and the crystallization of euhedral olivine with more fayalitic composition (e.g., Kurat et al. 1994; Engrand et al. 2005; Yada et al. 2005). Despite the macroscopic scale of meteorites, a comparison with cosmic spherules (<2 mm) is worthwhile, especially considering the occurrence of melt rim, similar to fusion crust, that is coating micrometeorites (Genge 2006), suggesting that even small objects can undergo extreme heating during the atmospheric entry. However, layering in the fusion crust was not observed to the same extent in cosmic spherules, suggesting that meteorite fusion crust experienced different cooling rates (strong thermal gradient) and recharge by new melt from the interior and from the distal part of the meteorite during the flight. These factors have likely affected the chemical evolution of the newly forming olivine in comparison with cosmic spherules, where complete melting and crystallization occurred at once, even though a certain extent of thermal gradient could have been developed also in some micrometeorites (Genge 2006). An effective comparison would be possible only after extensive investigation on a larger number of meteorite samples for fully constraining the processes. However, this will go beyond the aim of this work, dedicated to understanding the behavior of olivine in meteorite fusion crust in response to melting and reaction with the Earth's atmosphere.

## Conclusions

The fusion crusts of Antarctic ureilite A 09368 and H chondrites A 09004 and A 09502, all with particularly thick and extensively crystallized fusion crusts, have been investigated by focusing on the features shown by olivine crystallized from the melt.

1. In the fusion crust of the investigated ureilite and H chondrites (both representing relatively reduced starting materials), fast crystallization under relatively high *f*O_2_ conditions during entry into the Earth's atmosphere had strong effects on olivine morphology and composition.
2. The fusion crust appears layered in both meteorite types. This layering is reflected in olivine, outward from the contact with the thermally unaffected meteorite interior: (1) thermal fracturing and development of internal vesiculation, marked by enrichment in Mg/Fe ratio and depletion in highly volatile elements; (2) overgrowth of a more fosteritic rim; (3) overgrowth of fayalitic rim around relic magnesian cores and locally cyclical overgrowth resulting in oscillatory zoning; and finally (4), crystallization of hopper and dendritic olivine.
3. Variable cooling rates across the fusion crust occurred, as suggested by the variety of olivine shapes preserved. However, the cooling rate, i.e., the undercooling, is difficult to model in such a complex environment. Rough estimates based on the shape of olivine, according to Faure et al. (2003), are consistent with cooling rate of or higher than 2000 °C h^−1^.
4. Preferential crystallization of olivine and magnetite from the melt, which is not controlled by the original and local composition, can be explained by thermodynamic processes due to occurrence of oxidized Fe in the melt.
5. Change in secondary olivine composition during crystal growth, with an initial or repeated crystallization of more Mg‐rich olivine than the original material is likely due to (1) reductive conditions induced by C pyrolysis, i.e., in the case of the ureilite and as observed in carbonaceous chondrites and micrometeorites, and (2) removal of Fe by vaporization, as modeled for impact melts, followed by oxidation of Fe from di‐ to trivalent, leading to the formation of magnetite.
6. Detailed investigation of olivine, as presented in this work, provides constraints on cooling rate and redox reactions, which can be used for calibrating experiments reproducing the formation of meteorite fusion crust, for understanding observations in micrometeorites, and for improving numerical models on meteoroid atmospheric entry.

## Editorial Handling

Dr. Gretchen Benedix

## References

[maps13284-bib-0001] Bauer J. F . 1979 Experimental shock metamorphism of mono‐and polycrystalline olivine: A comparative study. Proceedings of the 10th Lunar and Planetary Science Conference, pp. 2573–2596.

[maps13284-bib-0002] Brandstätter F. , Brack A. , Baglioni P. , Cockell C. S. , Demets R. , Edwards H. G. M. , Kurat G. , Osinski G. R. , Pillinger J. M. , Roten C. A. , and Sancisi‐Frey S. 2008 Mineralogical alteration of artificial meteorites during atmospheric entry. The STONE‐5 experiment. Planetary and Space Science 56:976–984.

[maps13284-bib-0003] Donaldson C. H. 1976 An experimental investigation of olivine morphology. Contributions to Mineralogy and Petrology 57:187–213.

[maps13284-bib-0004] El Goresy A. and Fechtig H. 1967 Fusion crust of iron meteorites and mesosiderites and production of cosmic spherules. Smithsonian Contributions to Astrophysics 11:391–398.

[maps13284-bib-0005] Engrand C. , McKeegan K. D. , Leshin L. A. , Herzog G. F. , Schnabel C. , Nyquist L. E. , and Brownlee D. E. 2005 Isotopic compositions of oxygen, iron, chromium, and nickel in cosmic spherules: Toward a better comprehension of atmospheric entry heating effects. Geochimica et Cosmochimica Acta 69:5365–5385.

[maps13284-bib-0006] Falloon T. J. , Danyushev L. V. , Ariskin A. , Green D. H. , and Ford C. E. 2007 The application of olivine geothermometry to infer crystallization temperatures of parental liquids: implications for the temperature of MORB magmas. Chemical Geology 241:207–233.

[maps13284-bib-0007] Faure F. , Trolliard G. , Nicollet C. , and Montel J. M. 2003 A developmental model of olivine morphology as a function of the cooling rate and the degree of undercooling. Contributions to Mineralogy and Petrology 145:251–263.

[maps13284-bib-0008] Faure F. , Schiano P. , Troilliard G. , Nicollet C. , and Soulestin B. 2007 Textural evolution of polyhedral olivine experiencing rapid cooling rates. Contributions to Mineralogy and Petrology 153:405–416.

[maps13284-bib-0009] Genge M. J. 2000 Chondrule formation by the ablation of small planetesimals. Meteoritics & Planetary Science 35:1143–1150.

[maps13284-bib-0010] Genge M. J. 2006 Igneous rims on micrometeorites. Geochimica et Cosmochimica Acta 70:2603–2621.

[maps13284-bib-0011] Genge M. J. 2017 Vesicle dynamics during the atmospheric entry heating of cosmic spherules. Meteoritics & Planetary Science 52:443–457.

[maps13284-bib-0012] Genge M. J. and Grady M. M. 1999 The fusion crusts of stony meteorites: Implications for the atmospheric reprocessing of extraterrestrial materials. Meteoritics & Planetary Science 34:341–356.

[maps13284-bib-0013] Goodrich C. A. , Ash R. D. , van Orman J. A. , Domanik K. , and McDonough W. F. 2014 Metallic phases and siderophile elements in main group ureilites: Implications for ureilite petrogenesis. Geochimica et Cosmochimica Acta 112:340–373.

[maps13284-bib-0014] Hammer J. E. 2006 Influence of *f*(O)_2_ and cooling rate on the kinetics and energetics of Fe‐rich basalt crystallization. Earth and Planetary Science Letters 248:618–637.

[maps13284-bib-0015] Hezel D. C. , Dubrovinsky L. , Nasdala L. , Cauzid J. , Simionovici A. , Gellissen M. , and Schönbeck T. 2008 In situ micro‐Raman and X‐ray diffraction study of diamonds and petrology of the new ureilite UAE 001 from the United Arab Emirates. Meteoritics & Planetary Science 43:1127–1136.

[maps13284-bib-0016] Hezel D. C. , Poole G. M. , Hoyes J. , Coles B. J. , Unsworth C. , Albrecht N. , Smith C. , Rehkämper M. , Pack A. , Genge M. , and Russell S. 2015 Fe and O isotope composition of meteorite fusion crusts: Possible natural analogues to chondrule formation? Meteoritics & Planetary Science 50:229–242.

[maps13284-bib-0017] Horstmann M. , Humayun M. , Harries D. , Langenhorst F. , Chabot N. L. , Bischoff A. , and Zolensky M. E. 2013 Wüstite in the fusion crust of Almahata Sitta sulfide‐metal assemblage MS‐166: Evidence for oxygen in metallic melts. Meteoritics & Planetary Science 48:730–743.

[maps13284-bib-0018] Huss G. R. , Rubin A. E. , and Grossman J. N . 2006 Thermal metamorphism in chondrites In Meteorites and the Early solar system II, edited by Lauretta D. S. and McSween D. S. Jr Tucson, Arizona: University of Arizona Press pp. 567–586.

[maps13284-bib-0019] Hutchison R. 2004 Meteorites: A petrologic, chemical and isotopic synthesis. Oxford, UK: Cambridge University Press 506 p.

[maps13284-bib-0020] Jarosewich E. 1990 Chemical analyses of meteorites: A compilation of stony and iron meteorite analyses. Meteoritics 25:323–337.

[maps13284-bib-0021] Joy K. H. , Crawford I. A. , Downes H. , Russell S. S. , and Kearsley A. T. 2006 A petrological, mineralogical, and chemical analysis of the lunar mare basalt meteorite LaPaz Icefield 02205, 02224, and 02226. Meteoritics & Planetary Science 41:1003–1025.

[maps13284-bib-0022] Jurewicz A. J. G. , Mittlefehldt D. W. , and Jones J. H. 1991 Partial melting of the Allende (CV3) meteorite: Implications for the origins of basaltic meteorites. Science 252:695–698. 17746669 10.1126/science.252.5006.695

[maps13284-bib-0023] Kimura M. , Chen M. , Yoshida Y. , El Goresy A. , and Ohtani E. 2003 Back‐transformation of high‐pressure phases in a shock melt vein of an H‐chondrite during atmospheric passage: Implications for the survival of high‐pressure phases after decompression. Earth and Planetary Science Letters 217:141–150.

[maps13284-bib-0024] Koeberl C. and Hagen E. H. 1989 Extraterrestrial spherules in glacial sediment from the Transantarctic Mountains, Antarctica — Structure, mineralogy, and chemical composition. Geochimica et Cosmochimica Acta 53:937–944.

[maps13284-bib-0025] Kurat G. , Koeberl C. , Presper T. , Brandstätter F. , and Maurette M. 1994 Petrology and geochemistry of Antarctic micrometeorites. Geochimica et Cosmochimica Acta 58:3879–3904.

[maps13284-bib-0027] Lofgren G. and Lanier A. B. 1990 Dynamic crystallization study of barred olivine chondrules. Geochimica et Cosmochimica Acta 54:3537–3551.

[maps13284-bib-0028] Longhi J. 1999 Phase equilibrium constraints on angrite petrogenesis. Geochimica et Cosmochimica Acta 63:573–585.

[maps13284-bib-0029] Love S. G. and Brownlee D. E. 1994 Peak atmospheric entry temperatures of micrometeorites. Meteoritics 29:69–70.

[maps13284-bib-0030] Mittlefehldt D. W. , McCoy T. J. , Goodrich C. A. , and Kracher A . 1998 Non‐chondritic meteorites from asteroidal bodies In Planetary materials, edited by Papike J. J Reviews in Mineralogy, vol. 36. Washington, D.C: Mineralogical Society of America pp. 4‐001–4‐196.

[maps13284-bib-0031] Nagashima K. , Nara M. , and Matsuda J. 2012 Raman spectroscopic study of diamond and graphite in ureilites and the origin of diamonds. Meteoritics & Planetary Science 47:1728–1737.

[maps13284-bib-0032] Newcombe M. E. , Fabbrizio A. , Zhang Y. , Ma C. , Le Voyer M. , Guan Y. , Eiler J. M. , Saal A. E. , and Stolper E. M. 2014 Chemical zonation in olivine‐hosted melt inclusions. Contributions to Mineralogy and Petrology 168:1030 10.1007/s00410-014-1030-6.

[maps13284-bib-0033] Ni H. , Keppler H. , Walte N. , Schiavi F. , Chen Y. , Masotta M. , and Li Z . 2014 In situ observation of crystal growth in a basalt melt and the development of crystal size distribution in igneous rocks. Contributions to Mineralogy and Petrology 167:1003 (13 pp.). 10.1007/s00410-014-1003-9

[maps13284-bib-0034] Osako M. , Ito E. , and Yoneda A. 2004 Simultaneous measurements of thermal conductivity and thermal diffusivity for garnet and olivine under high pressure. Physics of the Earth and Planetary Interiors 143–144:311–320.

[maps13284-bib-0035] Putirka K. 2016 Rates and styles of planetary cooling on Earth, Moon, Mars, and Vesta, using new models for oxygen fugacity, ferric‐ferrous ratios, olivine‐liquid Fe‐Mg exchange, and mantle potential temperature. American Mineralogist 101:819–840.

[maps13284-bib-0036] Rubin A. E. , Fegley B. , and Brett R. 1988 Oxidation state in chondrites In Meteorites and the early solar system, edited by Kerridge J. F. and Matthew M. S. Tucson, Arizona: University of Arizona Press pp. 488–511.

[maps13284-bib-0037] Sheffer A. A. and Melosh H. J . 2005 A chemical model of micrometeorite impact into olivine. Abstract of the Workshop on Oxygen in Asteroids and Meteorites. Flagstaff, Arizona, USA. Abs. no. 7021.

[maps13284-bib-0038] Taylor S. and Brownlee D. E. 1991 Cosmic spherules in the geological record. Meteoritics 26:203–211.

[maps13284-bib-0039] Thaisen K. G. and Taylor L. A. 2009 Meteorite fusion crust variability. Meteoritics & Planetary Science 44:871–878.

[maps13284-bib-0040] Usui T. , Jones J. H. , and Mittlefehldt D. W. 2015 A partial melting study of an ordinary (H) chondrite composition with application to the unique achondrite Graves Nunataks 06128 and 06129. Meteoritics & Planetary Science 50:759–781.

[maps13284-bib-0041] Weisberg M. K. , McCoy T. J. , and Krot A. N. 2006 Systematics and evaluation of meteorite classification In Meteorites and the early solar system II, edited by Lauretta D. S. and McSween H. Y. Jr Tucson, Arizona: University of Arizona Press pp. 19–52.

[maps13284-bib-0042] Whitney D. L. and Evans B. W. 2010 Abbreviations for names of rock‐forming minerals. American Mineralogist 95:185–187.

[maps13284-bib-0043] Yada T. , Nakamura T. , Noguchi T. , Matsumoto N. , Kusakabe M. , Hiyagon H. , Ushikubo T. , Sugiura N. , Kojuma H. , and Takaoka N. 2005 Oxygen isotopic and chemical compositions of cosmic spherules collected from the Antarctic ice sheet: Implications for their precursor materials. Geochimica et Cosmochimica Acta 69:5789–5804.

[maps13284-bib-0044] Yamaguchi A. , Pittarello L. , Kimura M. , and Kojima H . 2014 Japanese/Belgian collection of Antarctic meteorites. Meteorite Newsletter 23. Tokyo, Japan: National Institute of Polar Research, Japan, 17 pp.

